# Editorial: Recent advances in our understanding of NEC pathogenesis, diagnosis, and treatment

**DOI:** 10.3389/fped.2023.1326204

**Published:** 2023-11-07

**Authors:** Misty Good, Minesh Khashu

**Affiliations:** ^1^ Division of Neonatal-Perinatal Medicine, Department of Pediatrics, University of North Carolina at Chapel Hill, Chapel Hill, NC, United States; ^2^ Neonatal Service, University Hospitals Dorset, Poole, United Kingdom

**Keywords:** necrotizing enterocolitis (NEC), artificial intelligence, breast milk, probiotics, neonate, intestine, NETs (neutrophil extracellular traps), preterm

**Editorial on the Research Topic** Recent advances in our understanding of NEC pathogenesis, diagnosis, and treatment

Necrotizing enterocolitis is a leading cause of death among premature infants, and despite research spanning over six decades, the pathogenesis is still not completely understood. The onset of NEC can be rapid and unpredictable, with clinical signs such as abdominal distension and bloody stools, progressing to fulminant bowel necrosis and death within hours. Even though the clinical and pathological descriptions of NEC were first described many decades ago, the management options have not progressed significantly and continue to be supportive care, such as cessation of feedings, intravenous fluids, antibiotic administration, and, in some cases, surgical bowel resection. Although treatment options for NEC remain limited, one effective preventative strategy is the administration of human milk. Recent advances in identifying the precise nutrients in human milk shed light on its bioactive components and their impact on the intestine. In recent years, several studies have highlighted the benefit of using prebiotics and probiotics as additional preventative options for NEC. Clinical studies focused on diagnostic tools such as using serum biomarkers or big data and artificial intelligence may pave the way for earlier detection to minimize disease progression, avoid the negative impact on other organ systems, and improve the poor neurodevelopmental outcomes associated with NEC. The primary objectives for this topic were to focus on recent advances in our understanding of NEC pathogenesis, new diagnostic strategies such as biomarkers and artificial intelligence, and explore new therapeutic options for treating this devastating disease.

This editorial highlights recent developments in the underlying pathogenesis of NEC, including the use of animal models, bench-to-bedside approaches, and machine-learning approaches for diagnostic purposes. This series of publications comprises state-of-the-art reviews, meta-analyses, and original research.

Singh et al. discuss bench-to-bedside approaches to understanding NEC pathogenesis, including a summary of the immunological status of infants with NEC and several defense mechanisms that become impaired in prematurity and NEC. The article by Klinke et al. describes the function of neutrophil extracellular traps (NETs) in necrotizing enterocolitis. NETs are released by neutrophils after contact with pathogens, and studies have shown that NET release is seen in mice and infants with NEC. This review discusses the various roles that NETs play in NEC, and specifically, that excessive NET formation can lead to hyperinflammation, contributing to disease pathogenesis. The manuscript by Bautista et al. is a state-of-the-art review describing the *in vivo* models of NEC. This review focuses on the descriptions of the different animal models, the phenotypic considerations, the strengths and weaknesses of each model, and how they recapitulate the human disease *in vivo*.

Sami et al. describe the role of human milk nutrients in preventing NEC. Preterm infants represent the most fragile population susceptible to developing NEC. Shortly after birth, their intestines face a series of challenges, including ongoing maturation, dietary demands driven by high nutritional needs, and the establishment of their gut microbial communities. Human milk is instrumental in shaping the gut microbiome, and this article summarizes the components of human milk, including lactoferrin, human milk oligosaccharides, dietary amino acids, vitamins, trace elements, and the interactions of these nutrients on the gut microbiota in NEC.

A review article of the current probiotic therapies for NEC by Sajankila et al. and a meta-analysis evaluating probiotics to prevent NEC in premature infants by Zhou et al. are helpful updates on this important aspect of preventing NEC. The review article discusses the various probiotic formulations, including single-strain formulations vs. multiple-strain formulations. In the meta-analysis, which included 27 randomized controlled trials with several different treatment interventions, they found that *Lactobacillus rhamnosus GG* and bovine lactoferrin can significantly reduce NEC incidence in preterm infants. While some questions are yet to be answered in terms of optimal probiotic combination and dosage, and there are concerns about sepsis related to non-medical grade probiotic use in premature infants, Sajankila et al. provided hope for the future with a discussion about the next generation of “designer probiotics,” which will need detailed study and evaluation.

Bethell et al. focus on recent advances surrounding NEC diagnosis, imaging modalities, and a discussion on the surgical approach to NEC. The state-of-the-art review on machine learning and artificial intelligence in NEC by McElroy and Lueschow explores using machine learning methods as a biomarker for NEC diagnosis, including using stool microbiome data and clinical demographics to predict infants at the highest risk for NEC. The limitations and pitfalls of our current use of machine learning and artificial intelligence should not dissuade us from utilizing these powerful tools for earlier diagnosis of NEC and improving outcomes.

Manohar et al. review the impact of the gut-brain axis on the long-term complications of NEC. NEC is associated with impaired long-term neurodevelopmental outcomes, including a higher incidence of cerebral palsy and cognitive deficits. The authors discuss the ways in which neurodevelopmental impairment is assessed, including cognitive developmental tests, as well as magnetic resonance imaging, and regions of the brain affected by NEC. This review discusses how the gut-brain axis plays a role in the neurodevelopmental impairment seen in NEC and how the microbiome, the innate immune system, and various neurotransmitters play a role in the pathogenesis of NEC-related neurodevelopmental impairment.

Early randomized controlled trials in the 1970s–1990s demonstrated that prophylactic antibiotics decreased the risk of NEC. However, more recent retrospective studies suggest prolonged antibiotic exposure is associated with increased risk for NEC. Cuna et al. discuss the use of early antibiotics and the risk of NEC in premature infants and mouse models of the disease, highlighting the mechanistic work evaluating the effects of early and prolonged antibiotic exposure in neonatal mice and piglets on the gut microbiome and intestinal immunity.

Finally, Mackay et al. report a pilot study using an untargeted aptamer-based proteomics assay as a biomarker discovery for NEC. They found ten serum proteins that could differentiate infants with NEC compared to controls with high sensitivity on a small sample volume. We look forward to further detailed study in this area.

This research topic has inspired significant discussions in the field of NEC research. Although more studies are desperately needed in this field, it is exciting that new developments are on the horizon.

